# Effects of the *MAML2* genetic variants in glioma susceptibility and prognosis

**DOI:** 10.1042/BSR20192091

**Published:** 2019-10-15

**Authors:** Ming Zhang, Yonglin Zhao, Junjie Zhao, Tingqin Huang, Xiaoye Guo, Xudong Ma, Yuan Wu

**Affiliations:** 1 Department of Neurosurgery, Second Affiliated Hospital of Xi’an Jiaotong University, Xi’an, Shaanxi 710004, China; 2 Department of Oncology, Second Affiliated Hospital of Xi’an Jiaotong University, Xi’an, Shaanxi 710004, China; 3 Department of Neurosurgery, First Affiliated Hospital of Xi’an Jiaotong University, Xi’an, Shaanxi 710061, China; 4 Department of Critical Care Medicine, Second Affiliated Hospital of Xi’an Jiaotong University, Xi’an, Shaanxi 710004, China

**Keywords:** Glioma, MAML2, prognosis, susceptibility

## Abstract

**Background:** Abnormal expression of the mastermind-like transcriptional co-activator 2 (*MAML2*) gene is oncogenic in several human cancers, including glioma. However, the relevance of *MAML2* variants with glioma remains unknown. We aimed to investigate the role of *MAML2* polymorphisms in glioma risk and prognosis among the Chinese Han population. **Methods:** Seven *MAML2* single-nucleotide polymorphisms (SNPs) were genotyped using Agena MassARRAY system among 575 patients with glioma and 500 age- and gender-matched healthy controls. Logistic regression was used to estimate the association between *MAML2* polymorphisms and glioma risk by calculating odds ratios (ORs) and 95% confidence intervals (CI). Kaplan–Meier survival analysis and univariate, multivariate Cox proportional hazard regression analyses for hazard ratios (HRs) and 95% CIs were performed to evaluate the contribution of *MAML2* polymorphisms to glioma prognosis. **Results:** *MAML2* rs7938889 and rs485842 polymorphisms were associated with the reduced risk of glioma (OR = 0.69, *P*=0.023; and OR = 0.81, *P*=0.032, respectively). Rs7115578 polymorphism had a lower susceptibility to glioma in males (OR = 0.68, *P*=0.034), while rs4598633 variant with a higher risk in females (OR = 1.66, *P*=0.016). Additionally, rs7115578 AG genotype represented a poorer prognosis of glioma (HR = 1.24, *P*=0.033) and astrocytoma (log-rank *P*=0.037, HR = 1.31, *P*=0.036). Furthermore, rs11021499 polymorphism had lower overall survival (OS) and progression-free survival (PFS) in patients with low-grade glioma. **Conclusion:** We provided some novel data suggesting *MAML2* polymorphisms might contribute to glioma risk and prognosis. Future studies are warranted to validate these findings and characterize mechanisms underlying these associations.

## Introduction

Glioma is one of the common types of primary central nervous system (CNS) tumors, accounting for 30% of all CNS tumors, almost 80% of which are considered malignant, and are responsible for the majority of deaths from primary brain tumors [1]. In 2015, the incidence and mortality of glioma in China were approximately 101600 and 61000, respectively, with a ratio of 3:2 for men and women [2]. The incidence of glioma in general increases with age, from 0.9 in children to 12.1 in the elderly [3]. Patients with glioma usually have poor survival rates and unfavorable prognosis. The etiology of glioma remains poorly understood and is attributed to different genetic or environmental factors [4]. Recently, a vast number of studies have reported that genetic factors contribute to the development of glioma, which revealed single-nucleotide polymorphisms (SNPs) in cancer-related genes were associated with glioma susceptibility and prognosis [5–7].

Mastermind-like transcriptional co-activator 2 (MAML2) is a member of the mastermind-like family of proteins, which is a co-activator of the oncogenic NOTCH signaling pathway [8]. NOTCH signaling activation has been demonstrated to be involved in carcinogenesis, which plays a critical role in cell proliferation, metastasis and epithelial–mesenchymal transition in many diverse solid tumors including glioma [9,10]. Several studies have demonstrated *MAML2* abnormal expression in various cancers, such as mucoepidermoid carcinoma, hidradenoma and breast cancer [11–13]. These studies have suggested that *MAML2* might be involved in the tumorigenesis and progression of cancers. Based on microarray data of glioma, *MAML2* as a novel gene related to glioma was identified [14]. Additionally, epidemiological studies have confirmed that polymorphisms in *MAML2*, a NOTCH pathway gene, were related to cancer susceptibility and prognosis [15,16]. However, no previous study has investigated the contribution of *MAML2* variants to glioma risk and prognosis.

In this hospital-based case–control study, we aimed to investigate the association of *MAML2* polymorphisms with the susceptibility of glioma, and the role of these polymorphisms in the prognosis of glioma patients among the Chinese Han population.

## Materials and methods

### Study subjects

This case–control study recruited 575 glioma patients and 500 cancer-free controls from the Second Affiliated Hospital of Xi’an Jiaotong University. All participants were genetically unrelated ethnic Han Chinese. Patients with primary glioma were newly diagnosed and histopathologically confirmed. The blood samples were collected before radiotherapy and chemotherapy therapies or surgery. The patients who had history of cancer and serious systemic diseases (leukemia, diabetes, cardiovascular and cerebrovascular diseases and genetic diseases) or other diseases were excluded. All the patients were followed up every 3 months. During the follow-up period, overall survival (OS) and progression-free survival (PFS) were measured from diagnosis to death or the last follow-up. The age and gender-matched healthy tumor-free volunteers were recruited from annual checkup visitors of the same hospitals as control subjects. The controls were selected from the skull MRI with a negative diagnosis for glioma, without other cancers or chronic diseases and no diseases related to brain and CNS. Demographic and clinical pathological data were collected through interviewers’ administered questionnaires and/or medical records. The institutional ethics committees of the Second Affiliated Hospital of Xi’an Jiaotong University approved the procedures followed in the present study. All procedures involving human participants were in accordance with the Helsinki Declaration. Signed informed consent was obtained from all individuals who participated in the study.

### SNPs genotyping

Genomic DNA was extracted from EDTA anticoagulated peripheral blood samples from each subject using a Qiagen DNA Isolation Kit (Qiagen, Valencia, CA, U.S.A.) according to the manufacturer’s instructions, and stored at −20°C until additional analysis. *MAML2* mRNA expression analysis in glioma was performed using GEPIA (http://gepia.cancer-pku.cn/) datasets. Seven *MAML2* SNPs (rs7107785, rs479825, rs7938889, rs11021499, rs7115578, rs4598633 and rs485842) were selected as candidate SNPs for genotyping in the current study. These SNPs were selected based on a minor allele frequency (MAF) of >5% in Chinese populations and with a pairwise *r^2^* ≥ 0.80, from the NCBI dbSNP database (http://www.ncbi.nlm.nih.gov/projects/SNP) and the 1000 Genomes Project data (http://www.internationalgenome.org/). To evaluate the potential function of the selected SNPs, we conducted *in silico* analysis using HaploReg v4.1 (https://pubs.broadinstitute.org/mammals/haploreg/haploreg.php) and SNPinfo Web Server (https://snpinfo.niehs.nih.gov/). *MAML2* SNPs genotyping was performed Agena MassARRAY system (Agena, San Diego, CA, U.S.A.) in double-blinded [17,18]. The primers used in amplification and single base extension were shown in Supplementary Table S1, which was designed by the MassARRAY Assay Design 3.0 software. For quality control, the call rate of genotyping >95%, and approximately 10% of the samples were randomly selected for repeated analysis, of which the reproducibility was 100%.

### Statistical analyses

All analyses were performed with the SPSS 18.0 (SPSS Institute, Chicago, IL, U.S.A.) and the PLINK 2.1.7 software. Baseline characteristics were presented as mean ± standard deviation (SD) for continuous data and as number (percentages) for categorical parameters. Variables were compared between the cases and controls using the Chi-squared for gender and the independent samples *t* test for age. Hardy–Weinberg equilibrium (HWE) was evaluated by Pearson’s χ^2^ test comparing the expected and observed genotype frequencies of *MAML2* SNPs in the control group. Differences in allele and genotype frequencies between glioma patients and cancer-free controls were also tested with χ^2^ test. The major allele was considered to be the reference allele. To determine the association between genotypes of *MAML2* SNPs and glioma risk, logistic regression analysis was performed to compute odds ratios (ORs) and 95% confidence intervals (CIs), with the adjustment of age and gender. Multiple genetic models (allele, genotype, dominant, recessive and log-additive) were estimated by PLINK software. Kaplan–Meier survival analysis with the log-rank test was used to evaluate the relationship between clinical or genomic factors (*MAML2* polymorphisms) and glioma prognosis. Hazard ratios (HRs) and 95% CIs were calculated from univariate and multivariate Cox proportional hazard regression analyses to evaluate the correlation between *MAML2* polymorphisms and glioma prognosis. All *P*-values were two-sided, and a *P*-value <0.05 was considered statistically significant.

## Results

### Subject characteristics

Demographic and clinical characteristics of glioma patients and controls are shown in Table 1. The patients included 320 males and 255 females with a mean age of 40.53 ± 13.90 years, and the controls included 279 males and 221 females with a mean age of 40.46 ± 18.08 years. No significant difference was observed in age and gender distribution between the two groups (*P*=0.942 and *P*=0.968, respectively). Among all cases, 369 (64.2%) were in grades I–II and 206 (35.8%) in III–IV and 448 (77.9%) with astrocytoma.

**Table 1:** Characteristics of patients with glioma and controls

	Characteristics	Cases (*n*=575)	Controls (*n*=500)	*P*
**Age, years**	Mean ± SD (year)	40.53 ± 13.90	40.46 ± 18.08	0.942^1^
**Gender**	Male	320 (55.7%)	279 (55.8%)	0.968^2^
	Female	255 (44.3%)	221 (44.2%)	
**WHO grade**	I–II	369 (64.2%)		
	III–IV	206 (35.8%)		
**Classification**	Astrocytoma	448 (77.9%)		
	Others	127 (22.1%)		
**Surgical method**	STR and NTR	183 (31.8%)		
	GTR	392 (68.2%)		
**Radiotherapy**	No	56 (9.7%)		
	Conformal radiotherapy	155 (27.0%)		
	γ knife	364 (63.3%)		
**Chemotherapy**	No	341 (58.8%)		
	Yes	237 (41.2%)		
**Survival condition**	Survival	40 (7.0%)		
	Lost	21 (3.6%)		
	Death	514 (89.4%)		

Abbreviations: GTR, gross-total resection; NTR, near-total resection; STR, subtotal resection; WHO, World Health Organization.

^1^*P*-values were calculated by independent samples *t* test.

^2^*P*-values were calculated by Chi-square tests.

### The details of MAML2 SNPs

Detailed information about the seven selected SNPs is displayed in Supplementary Table S2. Genotype distribution of all SNPs among controls was in agreement with HWE (*P*>0.05). *In silico* analysis using HaploReg v4.1 and SNPinfo Web Server, the function of the selected SNPs was successfully predicted to have biological functions (Supplementary Table S2). Furthermore, we extracted *MAML2* expression data between glioma patients and healthy controls from GEPIA database. Supplementary Figure S1 showed that there were significant differences of *MAML2* expression in glioblastoma multiforme and brain lower grade glioma compared with normal tissue (*P*<0.01). Moreover, the expression of *MAMAL2* was particularly associated with the prognosis of lower grade glioma (log-rank *P*=0.0094, Supplementary Figure S2).

### Association between MAML2 SNPs and glioma risk

Among the seven *MAML2* SNPs, two SNPs (rs7938889 and rs485842) were found to be significantly related to the risk of glioma by an adjustment for age and gender. The genotype and allele frequencies distribution of rs7938889 and rs485842 and their association with glioma risk are shown in Table 2. Subjects with rs7938889 TT genotype were associated with a reduced risk of glioma (TT vs. CC, OR = 0.69, 95%: 0.48–0.99, *P*=0.043; and TT vs. CC-CT, OR = 0.69, 95%: 0.50–0.95, *P*=0.023). Rs485842 polymorphism also displayed a lower risk of developing glioma under allele (OR = 0.81, 95%: 0.67–0.98, *P*=0.032), homozygote (OR = 0.59, 95%: 0.37–0.96, *P*=0.033) and additive (OR = 0.81, 95%: 0.67–0.98, *P*=0.033) models. Additionally, rs7938889 variant had a relationship with decreasing astrocytoma risk under multiple genetic model (allele, OR = 0.82, *P*=0.035; homozygote, OR = 0.61, *P*=0.013; recessive, OR = 0.63, *P*=0.009; and additive, OR = 0.81, *P*=0.027). No statistically significant associations were observed between *MAML2* rs7107785, rs479825, rs11021499, rs7115578 and rs4598633 variants and the risk of glioma.

**Table 2:** Relationships between *MAML2* polymorphisms and the risk of glioma and astrocytoma

SNP ID	Model	>Genotype	>Control (*n*)	Glioma			Astrocytoma		
				>*n*	>OR (95% CI)	>*P*	>*n*	>OR (95% CI)	>*P*
rs7938889	Allele	C	551	671	1		532	1	
		T	449	471	0.86 (0.73–1.02)	0.088	356	**0.82 (0.68–0.99)**	**0.035**
	Genotype	CC	150	183	1		148	1	
		CT	251	305	1.00 (0.76–1.31)	0.980	236	0.95 (0.71–1.26)	0.714
		TT	99	83	**0.69 (0.48–0.99)**	**0.043**	60	**0.61 (0.41–0.90)**	**0.013**
	Dominant	CC	150	183	1		148	1	
		CT-TT	350	388	0.91 (0.70**–**1.18)	0.473	296	0.85 (0.65**–**1.12)	0.252
	Recessive	CC-CT	401	488	1		384	1	
		TT	99	83	**0.69 (0.50–0.95)**	**0.023**	60	**0.63 (0.44–0.89)**	**0.009**
	Log-additive	**–**	**–**	**–**	0.85 (0.71**–**1.02)	0.080	**–**	**0.81 (0.67–0.98)**	**0.027**
rs485842	Allele	C	714	868	1		668	1	
		T	286	282	**0.81 (0.67–0.98)**	**0.032**	228	0.85 (0.70**–**1.04)	0.123
	Genotype	CC	258	326	1		250	1	
		CT	198	216	0.86 (0.67**–**1.11)	0.253	168	0.88 (0.67**–**1.15)	0.347
		TT	44	33	**0.59 (0.37–0.96)**	**0.033**	30	0.69 (0.42**–**1.13)	0.138
	Dominant	CC	258	326	1		250	1	
		CT-TT	242	249	0.81 (0.64**–**1.04)	0.094	198	0.84 (0.65**–**1.09)	0.192
	Recessive	CC-CT	456	542	1		418	1	
		TT	44	33	0.63 (0.39**–**1.01)	0.054	30	0.72 (0.45**–**1.18)	0.192
	Log-additive	**–**	**–**	**–**	**0.81 (0.67–0.98)**	**0.033**	**–**	0.85 (0.69**–**1.04)	0.116

*P*-values were calculated by logistic regression analysis with adjustments for age and gender.

*P*<0.05 means the data are statistically significant.

Bold means the data are statistically significant.

We also conducted stratification analyses by age and gender (Table 3), and revealed that the effect of both rs7938889 and rs485842 on glioma risk remained significant. The association between glioma risk and rs7938889 genotypes was more profound in the individuals at age ≤ 40 years (T vs C, OR = 0.75, *P*=0.020; TT vs CC, OR = 0.56, *P*=0.031) and males (TT vs CC, OR = 0.59, *P*=0.032), while rs485842 variant in the subjects at age > 40 years (T vs C, OR = 0.66, *P*=0.003; TT vs CC, OR = 0.31, *P*=0.0005). Furthermore, we also found that rs7115578 polymorphism had a lower susceptibility to glioma in males (AG-GG vs AA, OR = 0.68, *P*=0.034), while rs4598633 variant was associated with a higher risk for glioma in females (CT vs CC, OR = 1.66, *P*=0.016; CT-TT vs CC, OR = 1.49, *P*=0.043). We further stratified by the glioma grade, and there was a lower prevalence of rs7115578-GG carriers in the high-grade subgroup (WHO III+IV) than in the low-grade subgroup (WHO I+II) (16.99 vs 24.39%) with a marginal *P*-value in recessive model (OR = 0.64, 95% CI: 0.41–1.00, *P*=0.048, Supplementary Table S3), which indicated insufficient evidence for claiming an association and needed further verification.

**Table 3:** Relationships of *MAML2* polymorphisms with glioma risk stratified by age and gender

SNP ID	Model	Genotype	Case	Control	OR (95% CI)	*P*	Case	Control	OR (95% CI)	*P*
Age					>40				≤40	
rs7938889	Allele	C	330	264	1		341	287	1	
		T	254	206	0.99 (0.77–1.26)	0.913	217	243	**0.75 (0.59–0.96)**	**0.020**
	Genotype	CC	82	79	1		101	71	1	
		CT	166	106	1.44 (0.96–2.14)	0.075	139	145	**0.65 (0.44–0.96)**	**0.031**
		TT	44	50	0.83 (0.50–1.38)	0.473	39	49	**0.56 (0.33–0.95)**	**0.031**
	Dominant	CC	82	79	1		101	71	1	
		CT-TT	210	156	1.24 (0.85–1.80)	0.265	178	194	**0.63 (0.43–0.91)**	**0.014**
	Recessive	CC-CT	248	185	1		240	216	1	
		TT	44	50	0.66 (0.42–1.04)	0.073	39	49	0.73 (0.46–1.17)	0.189
	Log-additive	–	–	–	0.97 (0.75–1.25)	0.796	–	–	**0.73 (0.56–0.94)**	**0.016**
rs485842	Allele	C	458	325	1		410	389	1	
		T	134	145	**0.66 (0.50–0.86)**	**0.003**	148	141	1.00 (0.76–1.30)	0.976
	Genotype	CC	177	121	1		149	137	1	
		CT	104	83	0.85 (0.58–1.23)	0.378	112	115	0.93 (0.65–1.33)	0.682
		TT	15	31	**0.31 (0.16–0.59)**	**0.0005**	18	13	1.35 (0.63–2.89)	0.445
	Dominant	CC	177	121	1		149	137	1	
		CT-TT	119	144	**0.70 (0.49–0.99)**	**0.043**	130	128	0.97 (0.69–1.37)	0.866
	Recessive	CC-CT	281	204	1		261	252	1	
		TT	15	31	**0.33 (0.17–0.62)**	**0.001**	18	13	1.39 (0.66–2.94)	0.386
	Log-additive	–	–	–	**0.66 (0.50–0.86)**	**0.002**	–	–	1.03 (0.77–1.37)	0.844
**Gender**					**Male**				**Female**	
rs7938889	Allele	C	382	305	1		289	246	1	
		T	254	253	0.8 (0.64–1.01)	0.059	217	196	0.94 (0.73–1.22)	0.651
	Genotype	CC	107	83	1		76	67	1	
		CT	168	139	0.94 (0.65–1.35)	0.729	137	112	1.08 (0.71–1.63)	0.716
		TT	43	57	**0.59 (0.36–0.96)**	**0.032**	40	42	0.84 (0.49–1.45)	0.53
	Dominant	CC	107	83	1		76	67	1	
		CT-TT	211	196	0.84 (0.59–1.18)	0.309	177	154	1.01 (0.68–1.5)	0.945
	Recessive	CC-CT	275	222	1		213	179	1	
		TT	43	57	**0.61 (0.39–0.94)**	**0.025**	40	42	0.80 (0.5–1.29)	0.359
	Log-additive	–	–	–	0.79 (0.62–1.00)	0.054	–	–	0.94 (0.72–1.23)	0.641
rs7115578	Allele	A	365	289	1		262	245	1	
		G	275	269	0.81 (0.64–1.02)	0.069	248	197	1.18 (0.91–1.52)	0.211
	Genotype	AA	108	72	1		69	69	1	
		AG	149	145	0.68 (0.47–1.00)	0.048	124	107	1.16 (0.76–1.77)	0.495
		GG	63	62	0.68 (0.43–1.07)	0.097	62	45	1.38 (0.83–2.29)	0.217
	Dominant	AA	108	72	1		69	69	1	
		AG-GG	212	207	**0.68 (0.48–0.97)**	**0.034**	186	152	1.22 (0.82–1.82)	0.319
	Recessive	AA-AG	257	217	1		193	176	1	
		GG	63	62	0.86 (0.58–1.27)	0.447	62	45	1.26 (0.81–1.94)	0.302
	Log-additive	–	–	–	0.81 (0.65–1.02)	0.071	–	–	1.17 (0.91–1.51)	0.217
rs4598633	Allele	C	348	295	1		283	259	1	
		T	290	263	0.93 (0.74–1.17)	0.562	225	183	1.13 (0.87–1.46)	0.370
	Genotype	CC	96	74	1		71	81	1	
		CT	156	147	0.82 (0.56–1.19)	0.297	141	97	**1.66 (1.1–2.50)**	**0.016**
		TT	67	58	0.89 (0.56–1.42)	0.624	42	43	1.11 (0.66–1.9)	0.690
	Dominant	CC	96	74	1		71	81	1	
		CT-TT	223	205	0.84 (0.59–1.20)	0.334	183	140	**1.49 (1.01–2.20)**	**0.043**
	Recessive	CC-CT	252	221	1		212	178	1	
		TT	67	58	1.01 (0.68–1.50)	0.950	42	43	0.82 (0.51–1.31)	0.408
	Log-additive	—	—	—	0.93 (0.74–1.18)	0.557	—	—	1.13 (0.87–1.47)	0.364

*P*-values were calculated by logistic regression analysis with adjustments for age and gender.

*P*<0.05 means the data are statistically significant.

Bold means the data are statistically significant.

### Prognostic value of MAML2 SNPs in glioma patients

Of the 575 patients, 514 patients had complete follow-up data. We evaluated the impact of clinical factors on patients’ survival by Log-rank tests and univariate analysis, as shown in Supplementary Table S4 and Figure S3. The extent of resection (gross-total resection) was associated with the OS (log-rank *P*<0.001, HR = 0.63, *P*<0.001) and PFS (log-rank *P*<0.001, HR = 0.59, *P*<0.001) mortality hazards, respectively. Moreover, chemotherapy was a protective factor in all glioma patients (OS: log-rank *P*<0.001, HR = 0.67, *P*<0.001; PFS: log-rank *P*=0.012, HR = 0.81, *P*=0.025).

**Figure 1:**
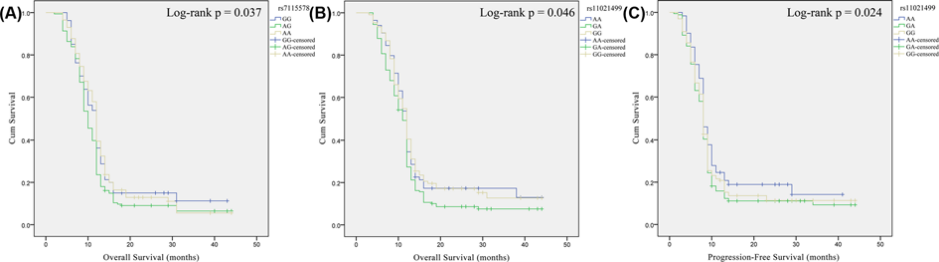
Kaplan–Meier survival curves for MAML2 polymorphism and glioma prognosis Kaplan–Meier survival curves for PFS based on *MAML2* rs7115578 in astrocytoma (**A**) and for OS and PFS based on *MAML2* rs11021499 polymorphism in low-grade glioma (**B,C**).

We investigated the association between *MAML2* polymorphisms and the prognosis of glioma patients using Log-rank tests and univariate Cox regression analysis (Table 4 and Figure 1). *MAML2* rs7115578 polymorphism was only one that affected the prognosis of glioma in the overall. Compared with the AA carriers of rs7115578, the AG genotype carriage represented a poorer prognosis of glioma (HR = 1.24, 95% CI: 1.02–1.52, *P*=0.033). In astrocytoma, rs7115578 polymorphism also was a predictor for unfavorable prognosis (OS: log-rank *P*=0.037, HR = 1.31, 95% CI: 1.02–1.69, *P*=0.036). We next divided all patients into two groups (low- and high-grade gliomas) according to WHO grade. In patients with low-grade glioma, Kaplan–Meier analyses and univariate analyses revealed that rs11021499-GA genotype had lower OS (log-rank *P*=0.046, HR = 1.30, 95% CI: 1.00–1.68, *P*=0.047) and PFS (log-rank *P*=0.024, HR = 1.33, 95% CI: 1.03–1.72, *P*=0.032) than CC genotype.

**Table 4:** Univariate analysis of the association between *MAML2* polymorphisms and glioma patient OS and PFS

SNP ID	Genotype	OS				PFS			
		Log-rank *P*	SR (1-/3-year)	HR (95% CI)	*P*	Log-rank *P*	SR (1-/3-year)	HR (95% CI)	*P*
**rs7115578**	AA	0.052	0.369/0.113	1		0.073	0.210/0.117	1	
	AG		0.276/0.071	**1.24 (1.02–1.52)**	**0.033**		0.160/0.075	1.22 (1.00–1.50)	0.051
	GG		0.336/0.111	1.07 (0.84–1.37)	0.595		0.185/-	1.06 (0.83–1.35)	0.661
**Astrocytoma**									
**rs7115578**	AA	**0.037**	0.395/0.055	1		0.093	0.206/0.072	1	
	AG		0.236/0.065	**1.31 (1.02–1.69)**	**0.036**		0.145/0.069	1.25 (0.97–1.61)	0.085
	GG		0.362/0.112	1.02 (0.75–1.38)	0.909		0.200/0.129	1.01 (0.74–1.37)	0.971
**Low-grade glioma (I–II)**									
**rs11021499**	GG	**0.046**	0.406/0.127	1		0.024	0.245/0.142	1	
	GA		0.274/0.075	**1.30 (1.00–1.68)**	**0.047**		0.147/-	**1.33 (1.03–1.72)**	**0.032**
	AA		0.345/-	1.02 (0.75–1.40)	0.885		0.214/-	1.02 (0.75–1.40)	0.883

Abbreviation: SR, survival rate.

Log-rank *P*-values were calculated using the Chi-Square test.

*P*<0.05 indicates statistical significance.

We next interrogated the association between *MAML2* SNPs and PFS or OS by a multivariate Cox proportional hazard model, adjusted for patient surgical method and chemotherapy (Table 5). The rs7115578 heterozygous was significantly associated with the poorer PFS of glioma (HR = 1.25, 95% CI: 1.02–1.53, *P*=0.031) and high-grade glioma (HR = 1.45, 95% CI: 1.03–2.03, *P*=0.032). Additionally, astrocytoma patients carrying the AG genotype had significantly decreased OS (HR = 1.40, 95% CI: 1.08–1.81, *P*=0.010) and PFS (HR = 1.38, 95% CI: 1.07–1.78, *P*=0.014) compared with those with the AA genotype.

**Table 5:** Multivariate analysis of the association between *MAML2* rs7115578 polymorphism and glioma patient OS and PFS

SNP ID	Genotype	OS		PFS	
		HR (95% CI)	*P*	HR (95% CI)	*P*
rs7115578	AA	1		1	
	AG	1.21 (1.00–1.49)	0.056	**1.25 (1.02–1.53)**	**0.031**
	GG	1.06 (0.83–1.36)	0.627	1.07 (0.84–1.37)	0.572
Astrocytoma					
rs7115578	AA	1		1	
	AG	**1.40 (1.08–1.81)**	**0.010**	**1.38 (1.07–1.78)**	**0.014**
	GG	1.17 (0.86–1.60)	0.306	1.19 (0.87–1.61)	0.275
High-grade glioma (III–IV)					
rs7115578	AA	1		1	
	AG	1.35 (0.96–1.89)	0.080	**1.45 (1.03–2.03)**	**0.032**
	GG	1.27 (0.81–1.98)	0.297	1.28 (0.82–1.99)	0.284

Log-rank *P*-values were calculated using the Chi-Square test.

*P*-values were calculated by Cox multivariate analysis with adjustments for surgical method and use of chemotherapy.

*P*<0.05 indicates statistical significance.

## Discussion

In the present study, we first investigated the association between *MAML2* genetic variants and glioma risk or prognosis among the Chinese Han population. We found that rs7938889, rs485842, rs7115578 and rs4598633 polymorphisms were significantly related to the risk of glioma. Moreover, we demonstrated that rs7115578 and rs11021499 variants represented a poorer prognosis of glioma. To the best of our knowledge, no previous studies have investigated the role of *MAML2* variants for glioma. Our study addressed a gap in knowledge of the *MAML2* gene polymorphisms and the susceptibility and prognosis of glioma, indicating that *MAML2* genetic variations might play an important role in the development of glioma.

MAML2, located at 11q21, normally acts as a co-activator of Notch receptor and transactivates Notch target gene, participating in the formation of Notch-associated RBP-J/CBF complex [19,20]. The oncogenic role of *MAML2* was first described in mucoepidermoid carcinoma, in which a fusion oncogene *MECT1-MAML2* that was involved in disrupting the normal cell cycle, differentiation and tumor development [21]. In addition, *MAML2* was previously found to participate in a fusion with *CRTC1*, which was important for cell growth, proliferation, survival, migration and metabolism [22]. These studies provided some biologic evidence for the role played by *MAML2* in possible molecular mechanisms of carcinogenesis. A recent study showed that the *CRTC1-MAML2* gene fusion was also identified in the brain tumors [23]. Additionally, *MAML2* as a novel gene was abnormal expressed in glioma [14]. By bioinformatics analysis, we found that *MAML2* gene expression is up-regulated in glioma compared with normal tissue. Moreover, low expression of *MAML2* was associated with a poor OS for glioma, especially lower grade glioma. These results hinted that *MAML2* plays an important role in the progression and prognosis of glioma, but more studies are needed to validate.

Previous studies have confirmed that the genetic variability of *MAML2*, including structural variation, copy number variation and SNPs, were associated with the occurrence and progression of disease [16,24,25]. In the present study, four SNPs in *MAML2* (rs7938889 and rs485842, rs7115578 and rs4598633) were found to be significantly associated with glioma risk. Specifically, carriers of the rs7938889 TT and rs485842 TT genotypes reduced the risk of the overall glioma and astrocytoma. Furthermore, the association between glioma risk and rs7938889 genotypes was more profound in the individuals at age ≤ 40 years, while rs485842 variant in the subjects at age > 40 years. We also found that rs7938889 and rs7115578 polymorphisms had a lower susceptibility to glioma in males, while rs4598633 variant was associated with a higher risk for glioma in females. There are differences of glioma incidence in gender and age [26]. This result suggested the risk association of *MAML2* polymorphisms with glioma might be dependent on age or gender. More importantly, we revealed that rs7115578 AG genotype represented a poorer prognosis of glioma, particularly among astrocytoma and high-grade glioma. Rs11021499 polymorphism had lower OS and PFS in patients with low-grade glioma. Although *MAML2* polymorphisms were found to be significantly associated with the risk and prognosis of glioma, the mechanism of *MAML2* underlying the effect on the glioma risk and patients survival was not identified in the present study, nor has not been reported in the literature. Several studies supported that intronic SNPs confer susceptibilities by affecting gene expression [27–29]. The online prediction tool Haploreg showed that rs7938889 and rs485842, rs7115578, rs4598633 and rs11021499 polymorphisms, located in the intron region, were associated with the regulation of promoter histone marks, enhancer histone marks, DNAse and/or motifs changed, suggesting their possible functions in glioma patients. However, further study is necessary to confirm the function of these variant in glioma.

Strengths of the current study include the relatively large sample size considering the rarity of glioma, and the first demonstration on the associations of *MAML2* polymorphisms with glioma risk and prognosis among Chinese Han population. However, several limitations should be addressed in the present study. First, the potential selection bias might have occurred because the study subjects in our study were hospital-based, thus the conclusion of the present study warrants further confirmation in a larger scale population. Second, the detailed molecular mechanism under which *MAML2* polymorphisms affect glioma risk and prognosis needs further studies to elucidate.

## Conclusion

In conclusion, these results suggested that *MAML2* polymorphisms might contribute to glioma susceptibility and prognosis. We found that *MAML2* rs7938889 and rs485842 polymorphisms were significantly associated with the reduced risk of glioma. Moreover, rs7115578 seem to confer a worse prognosis for glioma and astrocytoma. Although these data need confirmation by independent studies, our results hint *MAML2* genetic variants might play an important role in the development of glioma among Chinese Han population, and add to the body of knowledge surrounding genetic polymorphisms as putative player affecting the risk or prognosis of glioma.

## Informed Consent

Written informed consent was obtained from all of the subjects before participating.

## Supplementary Material

Supplementary Figures S1-S3 and Tables S1-S4

## Abbreviations

CI: confidence interval
CNS: central nervous system
HR: hazard ratio
HWE: Hardy–Weinberg equilibrium
MAML2: mastermind-like transcriptional co-activator 2
OR: odds ratio
OS: overall survival
PFS: progression-free survival
SNP: single-nucleotide polymorphism
