# Supplementary material for: Effects of the MAML2 genetic variants in glioma susceptibility and prognosis
Source: Biosci Rep. 2019 Oct 15;39(10):BSR20192091. doi: 10.1042/BSR20192091 (PMC6822528; doi:10.1042/BSR20192091)
Supplement: Supplementary Figures S1-S3 and Tables S1-S4 [file BSR-2019-2091_supp.pdf]

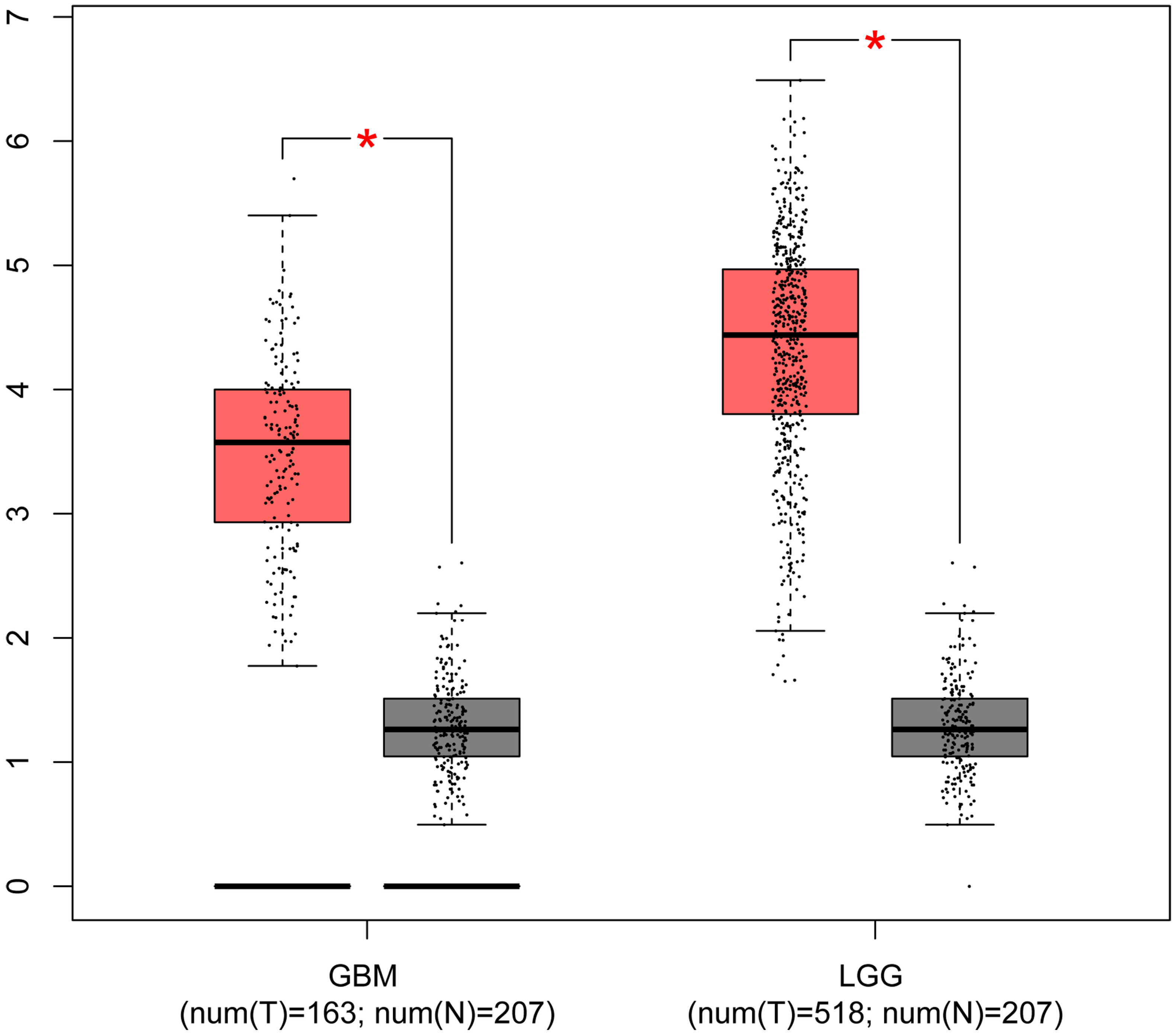

Supplementary Figure 1. MAML2 gene expression is up-regulated in glioma compared with that in normal lung tissues by GEPIA database.
GBM: glioblastoma multiforme; LGG lower grade glioma.
* indicates statistical significance ($p < 0.01$).

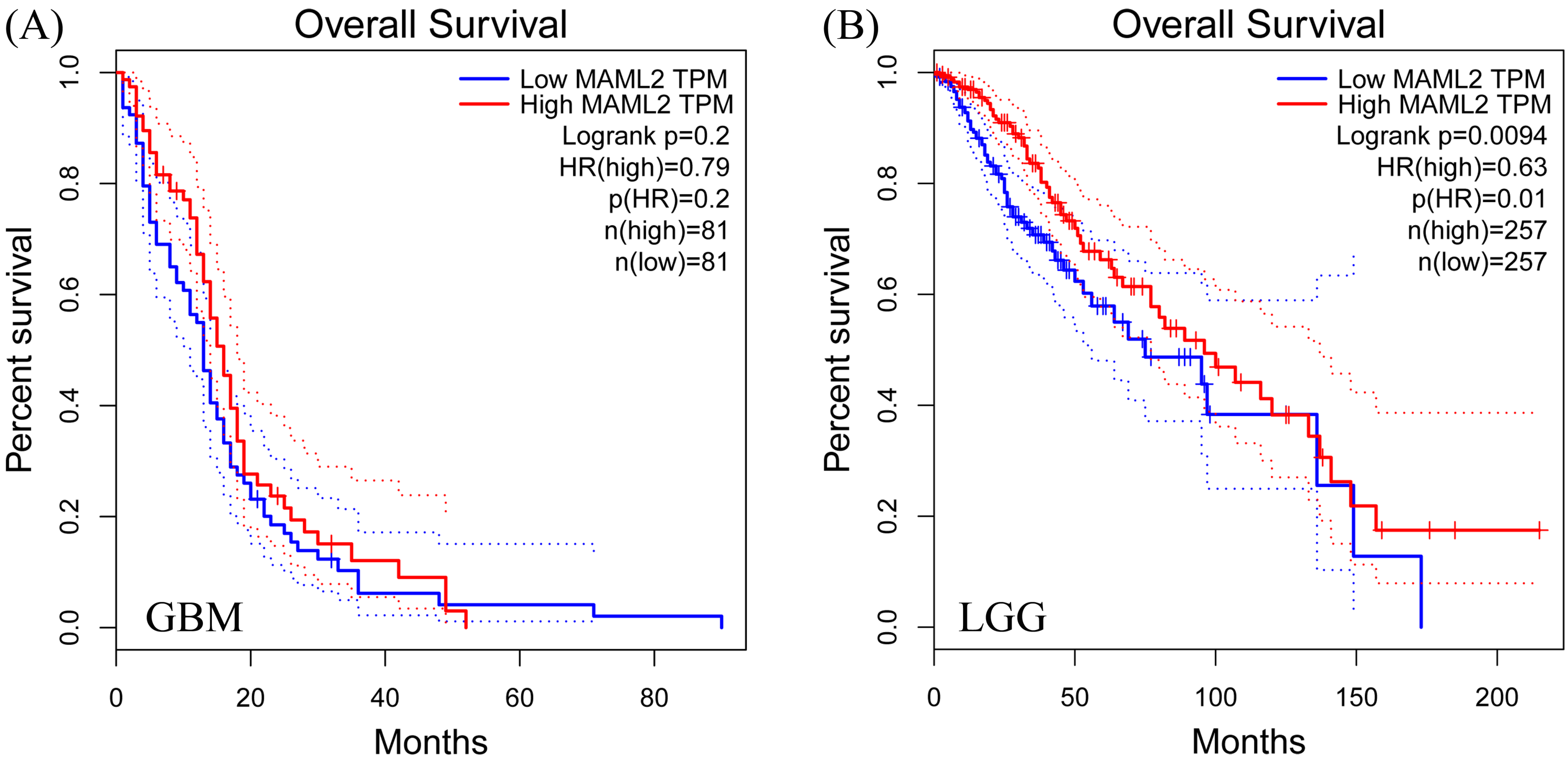

Supplementary Figure 2. Kaplan–Meier plots of overall survival for the MAML2 expression in glioma by GEPIA database.

GBM: glioblastoma multiforme; LGG lower grade glioma.

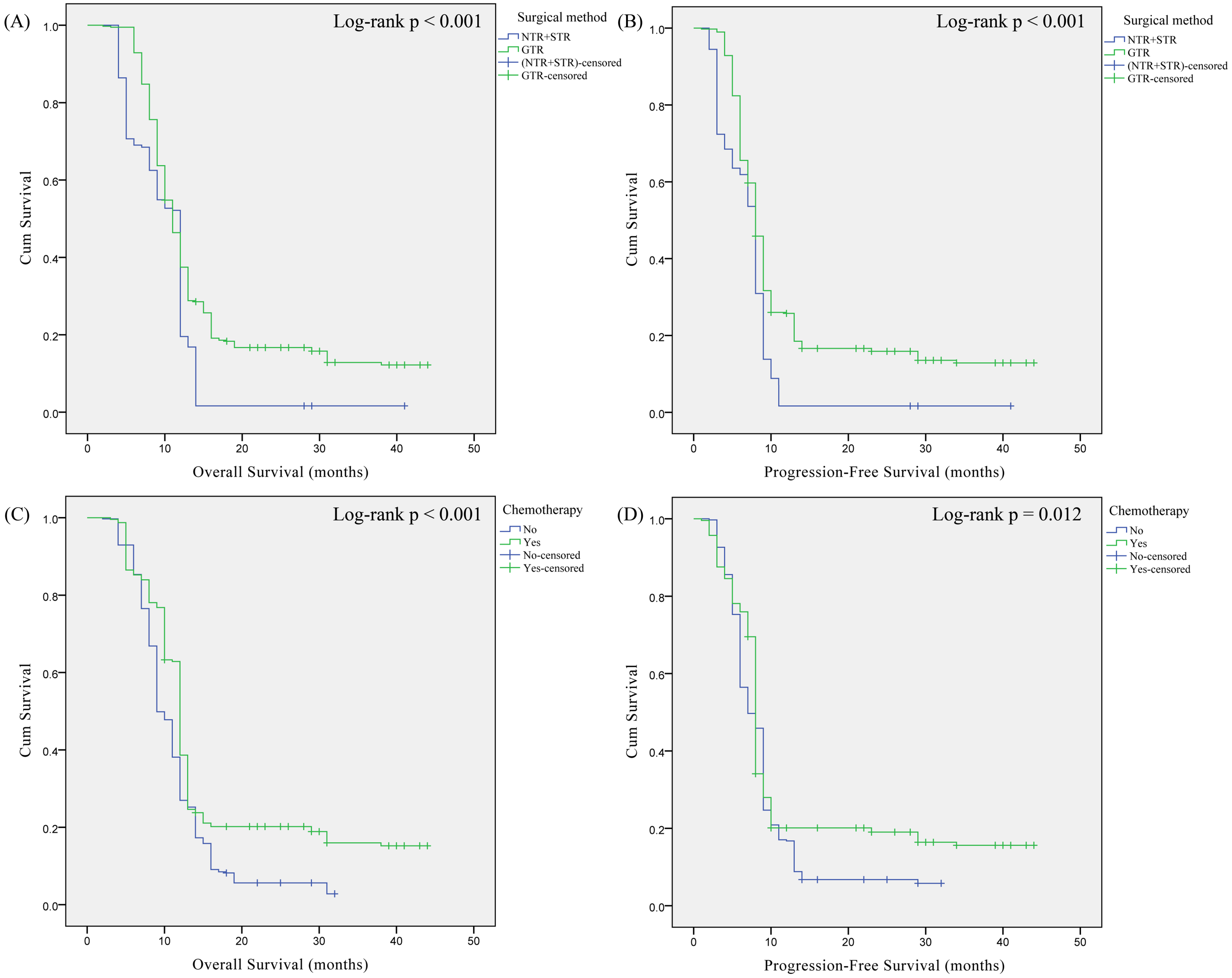

Supplementary Figure 3. Kaplan-Meier curves for overall survival and progression-free survival according to surgical method (A and C) and use of chemotherapy (B and D) in patients with glioma.

Supplementary Table 1. Primers sequence of PCR and UEP of *MAML2* SNPs used in this study

SNP	First Primer(5'-3')	Second Primer (5'-3')	UEP_DIR	UEP SEQ (5'-3')
rs7107785	ACGTTGGATGTGATGAAGCGAGCCACTTCC	ACGTTGGATGGAGTATACTGGGAGAATTGC	F	GGGACAAGGGAATCTTTC
rs479825	ACGTTGGATGATGCTCCTTTACGTGTTGCC	ACGTTGGATGCAAGCGATTCTGTGTGTTCC	F	CCTGAAGTTAGGCTCAC
rs7938889	ACGTTGGATGCAGAAAGGAAATGCGTTGTA	ACGTTGGATGCCCACATTTATGAAACAAGC	F	CCTATTAACTTGAATAATACTGAAATA
rs11021499	ACGTTGGATGAGAAGGGAGAGGACTTTCTG	ACGTTGGATGCTCAGGGCCTACAAAAAATG	F	gggtGAGCAACTTATTGTGGAG
rs7115578	ACGTTGGATGAATGAGCCCCTTCTGTGATG	ACGTTGGATGTGTATGATTTGCTCAATGGG	F	cGCTTAATAAAACATAACGTTATTGA
rs4598633	ACGTTGGATGTTTTTCCCCCTTAGTAAGAC	ACGTTGGATGAACAGCTTAGCTGCTATGAG	R	ACCACAATTGAAAAGCC
rs485842	ACGTTGGATGAGGCACCAGCCATTAAGAAG	ACGTTGGATGCTCTCTCACTTTTCTCTCAC	R	cTGTTCATTCTCTGCCAAC

SNP: single nucleotide polymorphism; UEP: unextended mini sequencing primer; DIR: direction; SEQ, sequence.

Supplementary Table 2. The information and HWE about the candidate SNPs in *MAML2*

SNP ID	Chr:Position	Role	Alleles (A/B)	MAF		*p*-value for HWE	Haploreg	SNPinfo Web Server
				Cases	Controls			
rs7107785	11:96014174	Intron	T/C	0.197	0.202	0.406	Promoter histone marks, Enhancer histone marks, DNAse, Proteins bound	TFBS
rs479825	11:96198321	Intron	C/T	0.339	0.353	0.493	Promoter histone marks, Enhancer histone marks, DNAse, Proteins bound, Motifs changed, NHGRI/EBI GWAS hits	
rs7938889	11:96231355	Intron	T/C	0.412	0.449	0.787	Promoter histone marks, Enhancer histone marks, Motifs changed	
rs11021499	11:96258965	Intron	G/A	0.468	0.491	0.422	Promoter histone marks, Enhancer histone marks, DNAse, Motifs changed, NHGRI/EBI GWAS hits	
rs7115578	11:96266936	Intron	G/A	0.455	0.466	0.857	Promoter histone marks, Enhancer histone marks, DNAse, Motifs changed, NHGRI/EBI GWAS hits	
rs4598633	11:96278187	Intron	T/C	0.449	0.446	0.786	Promoter histone marks, Enhancer histone marks, DNAse, GRASP QTLhits	
rs485842	11:96290050	Intron	T/C	0.245	0.286	0.511	Enhancer histone marks, Motifs change, NHGRI/EBI GWAS hits	

HWE, Hardy-Weinberg equilibrium; SNP, single nucleotide polymorphism; MAF, minor allele frequency; eQTL, expression quantitative trait loci; TFBS, transcription factor binding sites.

Supplementary Table 3. Relationship of clinical stage with *MAML2* rs7115578 polymorphism in glioma patients adjusted by WHO grade

SNP ID	Model	Genotype	III-IV	I-II	Adjusted by age and gender	
					**OR (95%CI)**	***p***
rs7115578	Allele	A	230	397	1	
		G	182	341	0.92 (0.72-1.17)	0.507
	Genotype	AA	59	118	1	
		AG	112	161	1.4 (0.94-2.08)	0.102
		GG	35	90	0.79 (0.48-1.31)	0.361
	Dominant	AA	59	118	1	
		AG-GG	147	251	1.18 (0.81-1.72)	0.390
	Recessive	AA-AG	171	279	1	
		GG	35	90	0.64 (0.41-1.00)	**0.048**
	Log-additive	---	---	---	0.93 (0.73-1.18)	0.559

SNP, single nucleotide polymorphism; OR, odds ratio; 95% CI, 95% confidence interval.

p values were calculated by logistic regression analysis with adjustments for age and gender.

$p < 0.05$ means the data is statistically significant.

Supplementary Table 4. Univariate analysis of the impact of clinical factors on glioma patient OS and PFS

Variables		Total	Event	OS					PFS				
				**Log-rank *p***	**MST (month)**	**SR (1-/3-year)**	**HR (95%CI)**	***p***	**Log-rank *p***	**MST (month)**	**SR (1-/3-year)**	**HR (95%CI)**	***p***
**Gender**	Male	320	284	0.352	14	0.326/0.088	1.08 (0.91-1.28)	0.394	0.241	12	0.203/0.097	1.10 (0.92-1.31)	0.293
	female	255	230		13	0.307/0.096				11	0.153/0.098		
**Age**	≤40	257	221	0.061	15	0.351/0.121	1.17 (0.98-1.39)	0.086	0.121	12	0.202/0.129	1.13 (0.95-1.35)	0.164
	>40	318	293		13	0.291/0.070				10	0.164/0.074		
**WHO grade**	Ⅰ-Ⅱ	369	324	0.094	14	0.328/0.111	1.15 (0.96-1.38)	0.125	0.122	12	0.191/0.118	1.14 (0.95-1.37)	0.166
	Ⅲ-Ⅳ	206	190		13	0.300/0.067				10	0.163/0.069		
**Surgical method**	NTR+STR	183	181	**<0.001**	10	0.196/-	**0.63 (0.52-0.76)**	**<0.001**	**<0.001**	7	0.017/-	**0.59 (0.49-0.71)**	**<0.001**
	GTR	392	333		15	0.375/0.129				13	0.258/0.135		
**Radiotherapy**	No	56	46	0.523	14	0.439/-	1.07 (0.94-1.22)	0.314	0.096	13	0.444/-	1.08 (0.95-1.24)	0.231
	Conformal radiotherapy	155	128		15	0.240/0.152				13	0.215/0.160		
	Gamma knife	364	340		13	0.332/0.060				10	0.165/0.064		
**Chemotherapy**	No	338	319	**<0.001**	11	0.270/0.028	**0.67 (0.56-0.81)**	**<0.001**	**0.012**	9	0.168/0.058	**0.81 (0.68-0.97)**	**0.025**
	Yes	237	195		16	0.387/0.152				14	0.201/0.164		

OS: Overall survival; PFS: Progression free survival; MST, median survival time, SR: Survival rate; HR: Hazard ratio; 95% CI: 95% Confidence interval.

NTR+STR: near-total resection & sub-total resection; GTR: gross-total resection

Log-rank p values were calculated using the Chi-Square test.

$p < 0.05$ indicates statistical significance.
